# Association Between Step Count Measured With a Smartphone App (Pain-Note) and Pain Level in Patients With Chronic Pain: Observational Study

**DOI:** 10.2196/23657

**Published:** 2022-04-06

**Authors:** Takahisa Ogawa, Luis Castelo-Branco, Kotaro Hatta, Chie Usui

**Affiliations:** 1 Department of Orthopedic Surgery Tokyo Medical and Dental University Tokyo Japan; 2 Neuromodulation Center Spaulding Rehabilitation Hospital Harvard Medical School Boston, MA United States; 3 Department of Psychiatry Juntendo University Nerima Hospital Juntendo University School of Medicine Tokyo Japan

**Keywords:** smartphone, iPhone, cross-sectional study, chronic pain, fibromyalgia, step count

## Abstract

**Background:**

Chronic pain is the leading cause of disability, affecting between 20% and 50% of the global population. The key recommended treatment is physical activity, which can be measured in daily life using a pedometer. However, poor adherence to pedometer use can result in incorrect measurements. Furthermore, only a few studies have investigated a possible curvilinear association between physical activity and chronic pain.

**Objective:**

In this study, we developed the Pain-Note smartphone app to collect real-world data on step count, using the smartphone’s built-in pedometer. The aims of our research are (1) to evaluate the association between daily step count and pain level among patients with chronic pain and (2) determine if the association between daily step count and pain level was curvilinear.

**Methods:**

We conducted a cross-sectional study based on step count data collected with the app and on the results of questionnaires, which measured the duration and intensity of pain, the widespread pain index, the symptom severity score, and the insomnia severity scale, including 7 questions for symptoms of depression. We analyzed the association between step count and pain level as a nonlinear relationship using a restricted cubic spline model. A prespecified subgroup analysis was also conducted based on fibromyalgia criteria.

**Results:**

Between June 1, 2018, and June 11, 2020, a total of 6138 records were identified, of which 1273 were analyzed. The mean age of the participants was 38.7 years, 81.9% (1043/1273) were female, and chronic pain was present for more than 5 years in 43.2% (550/1273) of participants. Participants in the third and fourth quartiles for step count (more than 3045 and 5668 steps a day, respectively) showed a significant positive association between higher step count and lower numerical pain rating scale (mean difference –0.43, 95% CI –0.78 to –0.08, *P*=.02; –0.45; 95% CI –0.8 to –0.1, *P*=.01, respectively) than those in the first quartile (less than or equal to 1199 steps a day). The restricted cubic spline model for the association between step count and pain scale displayed a steep decline followed by a moderate decrease as the step count increased; the inflection point was 5000 steps. However, this association was not observed among participants who met the fibromyalgia criteria (491/1273), who showed a steep positive increase below 2000 steps. Data were collected between June 1, 2018, and June 11, 2020, and were analyzed on November 18, 2021.

**Conclusions:**

Step count measured with the Pain-Note app showed a nonlinear association with pain level. Although participants with and without fibromyalgia showed a negative correlation between step count and pain level, participants who meet the criteria for fibromyalgia may present a different relationship between walking and pain perception compared to those in the general chronic pain population.

## Introduction

Chronic pain is a global health problem that affects from 20% to 50% of the general population, depending on the level of severity and how it is reported. It is also a leading cause of disability [1-5]. Low back and neck pain, osteoarthritis, and fibromyalgia are the major chronic musculoskeletal disorders associated with chronic pain. Chronic pain limits physical function and reduces the long-term quality of life. Health care costs associated with chronic pain range from US $261 to $300 billion per year in the United States, with total costs, direct and indirect, over $600 billion [6]. A previous randomized controlled trial suggested the use of nonpharmacological interventions, such as walking, to reduce pain and improve physical function [7]. In particular, increasing the level of daily activity in fibromyalgia has led to lower perceptions of functional deficits and pain level [8]. Since the mechanisms of pain perception are different in fibromyalgia and other types of chronic pain, the effectiveness of physical activity also differs with the type of chronic pain [9,10].

The US Centers for Disease Control and Prevention/American College of Sports Medicine guidelines recommend a minimum of 30 minutes per day of brisk walking for most adults [11], while the Ministry of Health, Labour and Welfare of Japan guidelines for health promotion advise Japanese adults to perform 60 minutes of moderate to vigorous physical activity per day [12] to achieve beneficial effects. In addition to exercise, previous research suggests that daily step count can be used to measure daily activity [13,14]. Pedometers are commonly used to measure total walking activity. However, use of pedometers is limited due to poor patient adherence; therefore, unless their use is effectively encouraged, the results can be an underestimate [15]. To study real-life daily activity with higher adherence, we used the pedometer function that is built into the iPhone smartphone platform (Apple Inc). To utilize the built-in pedometer function, we designed an app we named Pain-Note, which was based on the Research Kit function built into the iPhone.

One previous study analyzed data based on the assumption that the relationship between physical activity and pain is linear [16]. Some studies have found that the relationship between health care outcomes and physical activity is nonlinear when a restricted cubic spline model is used. [17-19] Therefore, we hypothesized that the association between step count and numerical pain rating scale would not be linear, as in previous studies that utilized a pedometer to measure physical activity. By using a built-in smartphone pedometer that allowed high adherence, we obtained data that reflected real-world circumstances. Further, an analysis based on a nonlinear model allowed us to assess the relationship between daily step count and pain.

This study aims to (1) evaluate the association between daily step count and pain level in patients with chronic pain, with consideration to the subtypes of chronic pain, using a pedometer developed on a smartphone platform; and to (2) determine if the association between daily step count and pain level was curvilinear.

## Methods

### Data Source and Participants

We conducted a cross-sectional study based on data collected with the Pain-Note app developed by Medical Logue Inc. (Tokyo, Japan). The company was under a consignment contract with the Faculty of Medicine of the Department of Psychiatry of our university.

The Pain-Note app was developed for subjects with chronic pain to help monitor their daily pain levels. It has been available in the Apple Store in Japan for free since June 1, 2018. This study included subjects who downloaded and used Pain-Note in Japan between June 1, 2018, and June 11, 2020, and completed the entire questionnaire. There was no financial compensation for participating in the study. Incomplete or duplicate answers were excluded.

### Data Collection

**Figure 1:**
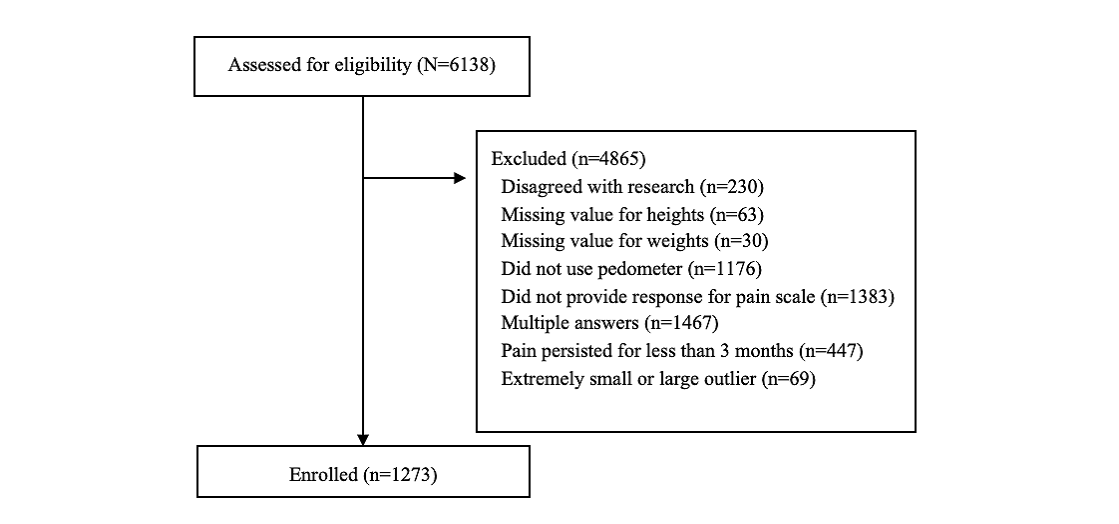
Flow chart of the study sample selection.

Pain-Note collected baseline characteristics, including demographic information, medical history, and lifestyle information. Disease-specific questionnaires were used to obtain data on the duration of pain, the widespread pain index (WPI) and symptom severity (SS) score for fibromyalgia, and the insomnia severity scale (ISS) for sleeping disorders. The questionnaire also included 7 questions on symptoms of depression. Participants also reported daily subjective symptoms, including baseline pain, which was recorded using a 10-point visual analog scale. After providing written consent, the study participants provided the following information in the following order in the Pain-Note app: baseline demographic characteristics, medical history (including cancer status and cardiac, respiratory, brain, liver, renal, hematological, and collagen disease), subjective pain symptoms, WPI, SS score, ISS, and symptoms of depression (Figure 1).

### Daily Step Count and Distance

We used the Research Kit function built into the iPhone to collect data on daily step count. The daily step count was automatically recorded in the participants’ smartphones for 24 hours a day, 365 days of the year, regardless of whether the Pain-Note app was running or not. We categorized participants into quartiles based on step count distribution on the day the participants answered the questionnaires. Previous research has categorized step count into quartiles to estimate its association with other baseline variables [20]. Participants in the 2.5th and 97.5th percentiles for step count were excluded as outliers.

### Chronic Pain Symptoms

Chronic pain data were collected using the WPI and SS score. WPI is a measure of pain or tenderness occurring within the 7 days before the test in 19 different body areas, from the jaws to the legs. The participants were also asked to rate their degree of physical pain on a scale of 0 to 19. The SS score measures the severity of symptoms on a scale of 0 to 12 by scoring fatigue, cognitive impairment, and unrefreshing sleep. Since the mechanisms of pain perception are different in fibromyalgia and other types of chronic pain, the effectiveness of physical activity also differs across types of chronic pain [9,10]. We determined the presence of fibromyalgia using the answers to the following questions in the questionnaire, based on the current fibromyalgia diagnosis guidelines: the duration of pain, WPI, and SS score [21]. The questions used to detect fibromyalgia symptoms included questions based on the validated Japanese version of the American College of Rheumatology 2010 criteria [22]. Based on answers from this section of the questionnaire, we classified patients into two groups: (1) those who fulfilled the criteria for fibromyalgia and (2) those who did not.

### Symptoms of Depression

Symptoms of depression were evaluated using 9 questions (Multimedia Appendix 1) that were based on depression research conducted in Japan and were consistent with other depression scales [23]. The association between depression and chronic pain is well established [24], as is the relationship between depression and low levels of physical activity [25].

### Symptoms of Sleep Disorder

Symptoms of sleep disorder were measured using the ISS, which assesses the severity of insomnia using 7 questions. The total score can range from 0 to 28 and indicates the severity of symptoms as follows: insomnia (0-7), subthreshold insomnia (8-14), and moderate and severe insomnia (15-28) [26]. The association between regular physical exercise and a lower incidence of sleep disturbance has been previously demonstrated [27]. In addition, sleep disturbance is common in chronic pain syndromes [28,29], especially fibromyalgia [29].

### Statistical Analyses

We compared patients’ baseline characteristics in all step count quartiles. Continuous variables are presented as the mean (SD) or median (IQR) based on their distribution. Categorical variables are presented as percentages. We conducted a 1-way ANOVA test for continuous variables and the chi-square test for categorical variables. We conducted multivariable regression analyses to identify and quantify the association between step count in each quartile and the pain scale; based on previous research, we adjusted for potential confounders, including age, sex, BMI, medical history (including cancer status and cardiac, respiratory, brain, liver, renal, hematological, and collagen disease), WPI, SS score, ISS, and depression questionnaire answers [23].

A previous study [16] suggested that there cannot be a linear relationship between physical activity, including step count, and clinical outcomes, such as pain level, in fibromyalgia. In response, we performed a restricted cubic spline analysis, which is able to investigate nonlinear relationships between 2 continuous variables. We followed the rationale reported by Marrie et al [30] and placed the knots, which are breakpoints representing inflections in the distribution of the data, at the 5th, 35th, 65th, and 95th percentiles, to make the model flexible enough for our assumed nonlinear association. We evaluated nonlinearity using the likelihood-ratio test by comparing the model fit when cubic spline terms were used and when only linear terms were used [31]. To test the association between fibromyalgia and the number of steps, we also constructed linear regression models that included fibromyalgia symptoms as the independent variable and step count as the dependent variable. We then adjusted for potential confounders, including age, sex, pain level, ISS, and symptoms of depression.

### Sensitivity Analysis

We conducted a sensitivity analysis to confirm the robustness of the main results. We used the daily distance moved, which was measured using the built-in smartphone GPS (rather than the step count) to confirm the relationship between distance moved and pain scale score. All comparison tests were 2-sided and set statistical significance as *P<*.05. We did not adjust the significance level for multiple comparisons due to the exploratory nature of our study. All statistical analyses were performed using Stata (version 16.1; Stata Corp).

### Ethics Approval

This study was conducted after obtaining approval from the independent ethics committee of our university hospital. Written informed consent was obtained electronically from all participants before they answered the questionnaires. Ethical approval was obtained from the Research Ethical Committee of Juntendo University Nerima Hospital on March 3, 2020 (2019032). Data were collected between June 1, 2018, and June 11, 2020, and analyzed on November 18, 2021.

## Results

**Figure 2:**
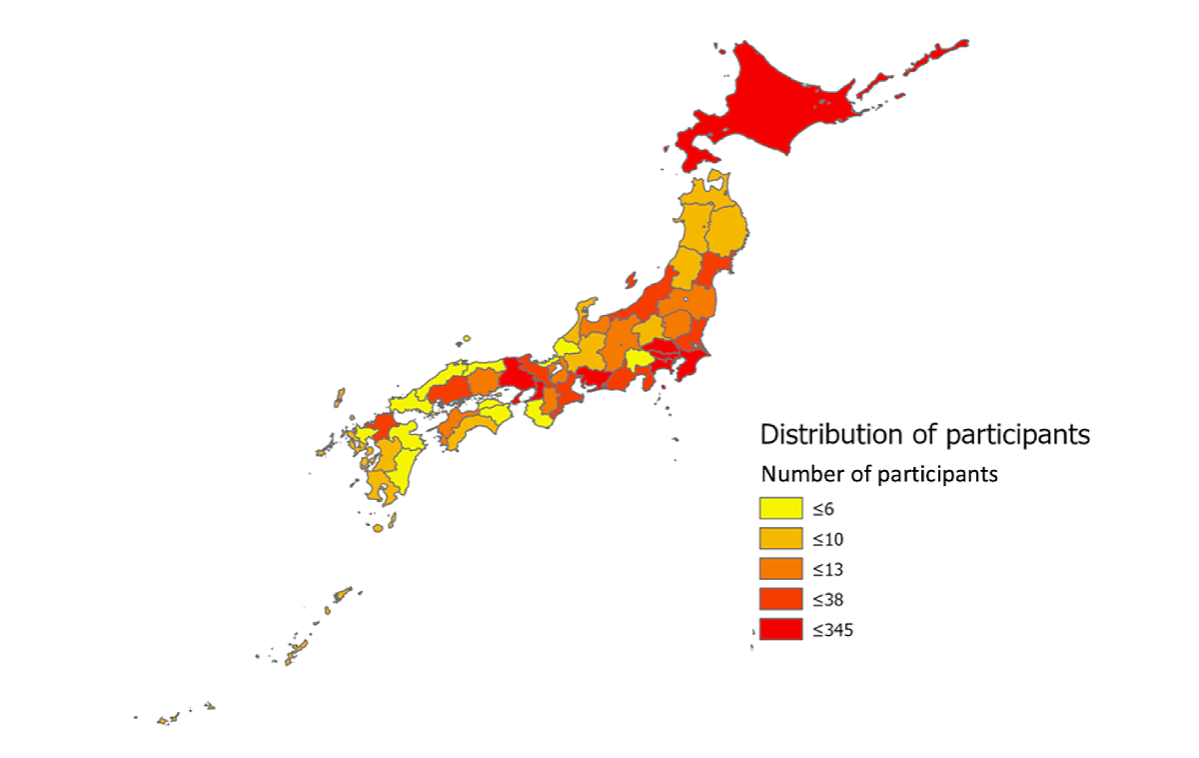
Geographic distribution of participants.

A total of 6138 records were identified in our database. A total of 4865 records were excluded for the following reasons: consent was not provided for the research (n=230), height data were missing (n=63), weight data were missing (n=30), the pedometer function was disabled (n=1176), pain scale data were lacking (n=1383), data were duplicated (n=1467), pain history was less than 3 month (n=447), or the record was an extremely small or large outlier (n=69). A total of 1273 participants were included in the study (Figure 1). The mean age was 38.7 years (SD 13.4), and the number of women was 1043 of 1273 (81.9%). The participants mostly lived in large cities in Japan, such as Tokyo, Nagoya, Osaka, and Sapporo (Figure 2).

Table 1 shows the baseline characteristics of the participants. Participants in the first quartile had 24 to 1199 steps, the second quartile, 1205 to 3036 steps, the third quartile, 3045 to 5664 steps, and the fourth quartile, 5668 to 14,473 steps. Participants in the first quartile had the highest mean age at 40.22 years (SD 13.75) and the highest BMI at 23.8 (SD 5.6), while those in the third quartile had the lowest mean age at 36.97 years (SD 13.53) and those in the fourth quartile had the lowest BMI at 22.8 (SD 4.4). Among comorbidities, the largest proportion of participants had respiratory disease (210/1273, 16.5%). Among the 210 participants with respiratory disease, participants in the first quartile were the most numerous, followed by patients in the third, fourth, and second quartiles (66/210, 21%; 57/210, 18%; 44/210, 14%; and 43/210, 14%, respectively).

**Table 1:** Patient characteristics.

Characteristics		Total	First quartile (24-1199 steps)	Second quartile (1205-3036 steps)	Third quartile (3045-5664 steps)	Fourth quartile (5668-14,473 steps)	*P* value^a^
Age (years), mean (SD)		38.72 (13.47)	40.22 (13.75)	39.53 (12.77)	36.97 (13.53)	38.14 (13.65)	.01
Female sex, n (%)		1043 (81.9)	281 (88.1)	265 (83.3)	260 (81.8)	237 (74.5)	<.001
BMI, (kg/m^2^), mean (SD)		23.2 (4.9)	23.8 (5.6)	23.3 (5)	22.9 (4.7)	22.8 (4.4)	.04
**Smoking**							.30
	Nonsmoker, n (%)	844 (66.3)	210 (65.8)	205 (64.5)	217 (68.2)	212 (66.7)	
	Current smoker, n (%)	178 (14)	45 (14.1)	50 (15.7)	49 (15.4)	34 (10.7)	
	Past smoker, n (%)	251 (19.7)	64 (20.1)	63 (19.8)	52 (16.4)	72 (22.6)	
Heart disease, n (%)		78 (6.1)	16 (5)	19 (6)	23 (7.2)	20 (6.3)	.71
Respiratory disease, n (%)		210 (16.5)	66 (20.7)	43 (13.5)	57 (17.9)	44 (13.8)	.04
Stroke, n (%)		44 (3.5)	17 (5.3)	12 (3.8)	8 (2.5)	7 (2.2)	.12
Liver disease, n (%)		45 (3.5)	14 (4.4)	14 (4.4)	7 (2.2)	10 (3.1)	.36
Renal disease, n (%)		59 (4.6)	15 (4.7)	16 (5)	12 (3.8)	16 (5)	.86
Hematological disease, n (%)		37 (2.9)	12 (3.8)	6 (1.9)	13 (4.1)	6 (1.9)	.19
Cancer, n (%)		42 (3.3)	11 (3.4)	10 (3.1)	10 (3.1)	11 (3.5)	.99
Collagen disease, n (%)		91 (7.1)	21 (6.6)	25 (7.9)	23 (7.2)	22 (6.9)	.93

^a^*P* was calculated with ANOVA for age and BMI and the chi-square test for all other variables.

Data on pain level, depression, and sleeping disorders are shown in Table 2. More than 40% (550/1273) of the participants had chronic pain for at least 5 years. The second quartile had the fewest participants with chronic pain lasting more than 5 years (122/318, 38%). The second quartile had the largest number of participants with clinical insomnia (62/318, 20%). Participants in the first quartile had the most symptoms of depression, while those in the fourth quartile had the least.

**Table 2:** Step count and findings on pain, sleeping disorders, and responses to depression questionnaire.

				Total group	First quartile for step count	Second quartile for step count	Third quartile for step count	Fourth quartile for step count	*P* value^a^
Step count, median (IQR)				3036.0 (1199.0-5664.0)	324.0 (95.0-636.0)	1995.5 (1557.0-2454.0)	4281.5 (3623.0-4927.0)	7998.5 (6602.0-10,050.0)	
**Chronic pain, n (%)**									.009
			Current	1180 (92.7)	307 (96.2)	298 (93.7)	289 (90.9)	286 (89.9)	
			Past	93 (7.3)	12 (3.8)	20 (6.3)	29 (9.1)	32 (10.1)	
**Duration of pain, n (%)**									.52
			3-6 months	140 (11)	38 (11.9)	30 (9.4)	35 (11)	37 (11.6)	
			6-12 months	118 (9.3)	25 (7.8)	32 (10.1)	28 (8.8)	33 (10.4)	
			1-2 years	254 (20)	58 (18.2)	69 (21.7)	66 (20.8)	61 (19.2)	
			3-5 years	211 (16.6)	55 (17.2)	65 (20.4)	48 (15.1)	43 (13.5)	
			>5 years	550 (43.2)	143 (44.8)	122 (38.4)	141 (44.3)	144 (45.3)	
**Presence of fibromyalgia and severity of symptoms**									
		Fibromyalgia, n (%)		491 (38.6)	160 (50.2)	131 (41.2)	112 (35.2)	88 (27.7)	<.001
	Widespread pain index, mean (SD)			8.6 (6.7)	9.9 (6.8)	9.2 (6.6)	7.9 (6.7)	7.2 (6.6)	<.001
	Symptom severity score, mean (SD)			4.1 (2.3)	4.6 (2.3)	4.1 (2.4)	4.0 (2.2)	3.8 (2.3)	<.001
**Insomnia severity scale, n (%)**									<.001
			Not clinically significant	536 (42.1)	96 (30.1)	139 (43.7)	142 (44.7)	159 (50)	
			Subthreshold insomnia	521 (40.9)	172 (53.9)	117 (36.8)	123 (38.7)	109 (34.3)	
			Clinical insomnia	216 (17.0)	51 (16)	62 (19.5)	53 (16.7)	50 (15.7)	
**Questionnaire for depression, n (%)^b^**									
			Do you enjoy your life?	696 (58.2)	210 (68.2)	165 (56.7)	172 (58.5)	149 (49.3)	<.001
			Do you enjoy things the same way that you used to?	683 (57.2)	209 (67.9)	172 (59.1)	155 (52.7)	147 (48.7)	<.001
			Do you feel tired when doing things you could easily do before?	254 (21.3)	49 (15.9)	56 (19.2)	59 (20.1)	90 (29.8)	<.001
			Do you feel as well as other people?	664 (55.6)	187 (60.7)	142 (48.8)	169 (57.5)	166 (55)	.03
			Do you think about death?	555 (46.4)	133 (43.2)	136 (46.7)	135 (45.9)	151 (50.0)	.41
			Do you feel so depressed that you think about suicide?	743 (62.2)	184 (59.7)	181 (62.2)	177 (60.2)	201 (66.6)	.29
			Recently, do you find things very difficult or painful?	339 (28.4)	66 (21.4)	74 (25.4)	85 (28.9)	114 (37.7)	<.001
			Do you have a good appetite?	689 (57.7)	159 (51.6)	171 (58.8)	176 (59.9)	183 (60.6)	.09
			Do you feel depressed?	269 (22.5)	57 (18.5)	60 (20.6)	71 (24.1)	81 (26.8)	.07

^a^All *P* values were calculated with the chi-square test.

^b^Data for depression represent “yes” answers on the questionnaire.

**Figure 3:**
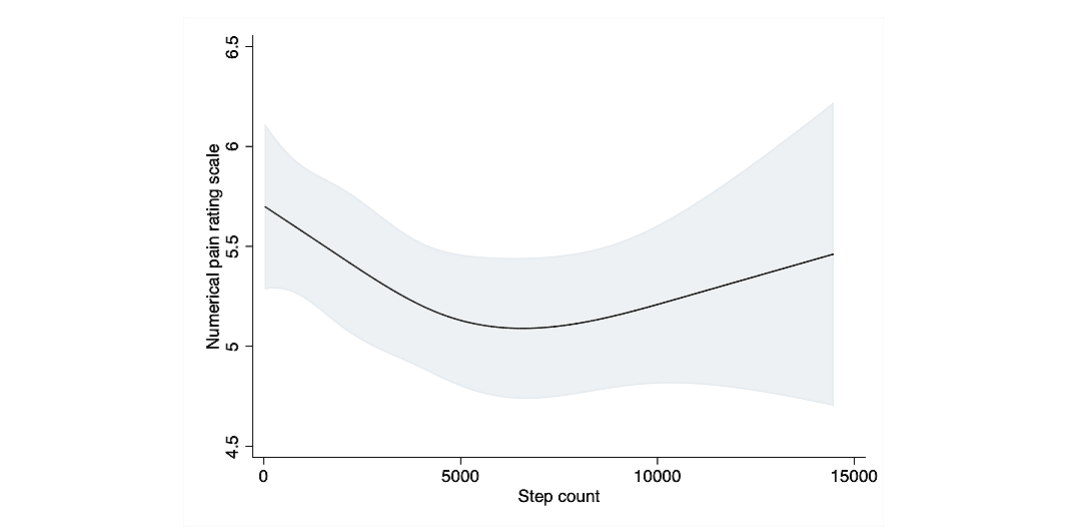
Association between step count and pain scale using restricted cubic splines. Four knots restricted the cubic spline curve. The curve showed an inflection point around 5000 steps.

**Figure 4:**
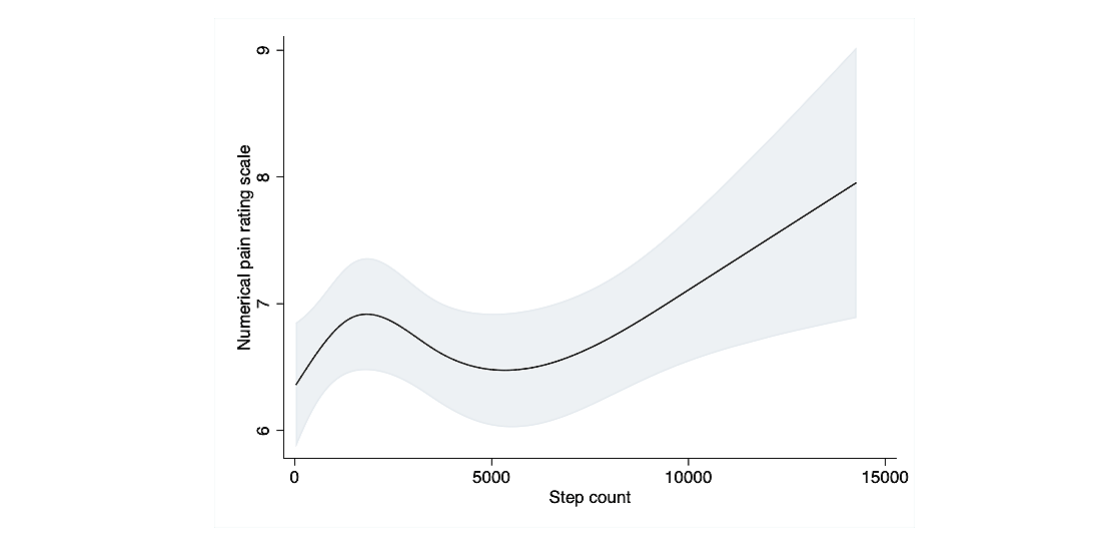
Association between distance and pain scale using restricted cubic splines in fibromyalgia patients. Four knots restricted the cubic spline curve. The curve showed an initial peak in numerical pain rating scale around 2000 steps and started to increase after 5000 steps.

**Figure 5:**
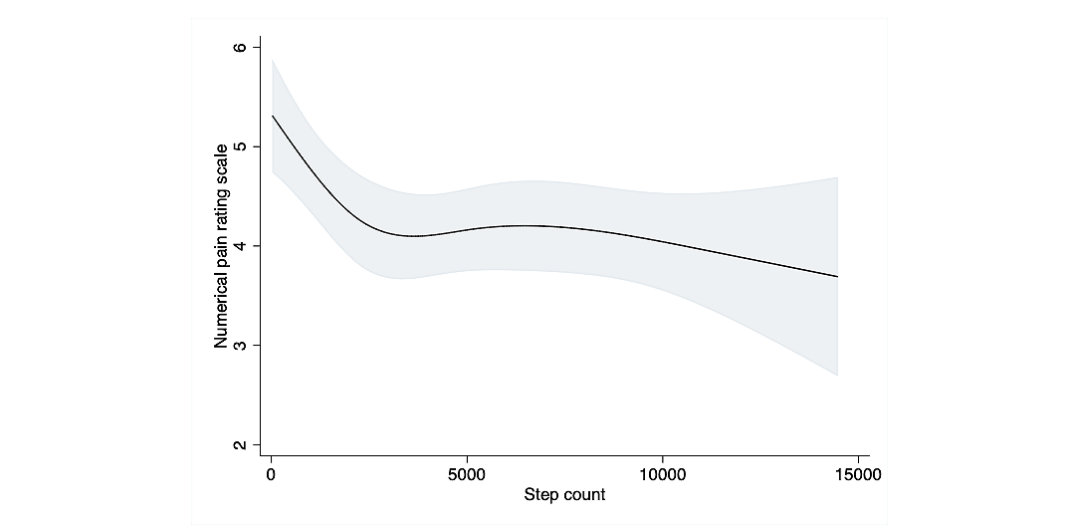
Association between distance and pain scale using restricted cubic splines in nonfibromyalgia patients. Four knots restricted the cubic spline curve. Step count and numerical pain rating scale were negatively correlated before 3000 steps, then showed no association past 3000 steps.

A restricted cubic splines model was used to assess the association between step count and pain scale; it revealed an obvious nonlinear association in the overall sample (Figure 3). When compared to the results of the linear regression, the results of the likelihood test were nearly, but not quite, statistically significant, which suggests that a model including patients with and without fibromyalgia would accommodate a nonlinear relationship with a better fit than a linear model for the association between step count and pain scale (*P=*.06). The results of the multivariable linear regression model for pain scale (divided in quartiles) are shown in Table 3. Participants in the third and fourth quartiles, who had a higher step count, were significantly less likely to report pain (mean difference –0.43, 95% CI –0.78 to –0.08, *P=*.02; mean difference –0.45, 95% CI –0.8 to –0.1, *P=*.013, respectively). Stratifying the sample into 2 groups based on the fibromyalgia criteria revealed no significant association between step count and pain scale score in any of the 4 quartiles in the fibromyalgia group. However, the restricted cubic spline curve in the fibromyalgia group showed a mild increase in pain level as step count increased, until an inflection point around 2000 daily steps, followed by a decrease between 2000 and 5000 steps, suggesting the importance of visualization with the restricted cubic spline curve. The nonfibromyalgia group showed a steep negative association between step count and pain until around 3000 daily steps, followed by a nearly unchanging, steady line above that (Figure 4, Figure 5). Among participants with chronic pain but without fibromyalgia, the second, third, and fourth step count quartiles demonstrated a significant linear association between step count and reduced pain (mean difference –0.61, –0.59, and –0.78, *P=*.01, .02, and .002, respectively) (Table 3).

**Table 3:** Association between step count by quartile and pain scale in a multivariable regression model.^a^

Type of pain		Mean difference (95% CI)	*P* value
**Overall chronic pain**			
	1st quartile (24-1199 steps)	Reference	
	2nd quartile (1205-3036 steps)	–0.19 (–0.54 to 0.16)	.29
	3rd quartile (3045-5664 steps)	–0.43 (–0.78 to –0.08)	.02
	4th quartile (5668-14,473 steps)	–0.45 (–0.8 to –0.1)	.01
**Participants fulfilling fibromyalgia criteria**			
	1st quartile (25-657 steps)	Reference	
	2nd quartile (662-2187 steps)	0.26 (–0.23 to 0.74)	.30
	3rd quartile (2188-4578 steps)	0.29 (–0.2 to 0.78)	.25
	4th quartile (4600-14,264 steps)	0.17 (–0.33 to 0.66)	.51
**Participants not fulfilling fibromyalgia criteria**			
	1st quartile (24-1396 steps)	Reference	
	2nd quartile (1397-3537 steps)	–0.61 (–1.09 to –0.12)	.01
	3rd quartile (3538-6198 steps)	–0.59 (–1.08 to –0.1)	.02
	4th quartile (6199-14,473 steps)	–0.78 (–1.27 to –0.29)	.002

^a^All models were adjusted for age, sex, and comorbidities that included cardiological, respiratory, stroke, liver, renal, and hematological disease, cancer, symptom severity scale, insomnia scale, and the results of the depression questionnaire.

As the spline curves suggested a curvilinear association, the likelihood test for both restricted cubic spline curve models (ie, patients with fibromyalgia and those without) showed that the fit was statistically significantly superior to that of a linear regression model (*P=*.03 and *P=*.007, respectively) when the data were stratified for the presence of fibromyalgia, while in the overall model, the difference was not significant (*P*=.06). This suggests that the relationship between step count and pain scale should be considered separately in patients with and without fibromyalgia. In our sensitivity analysis, the association between distance and pain scale was similar to our main analysis.

The results of the univariate linear regression model revealed a statistically significant association between the presence of fibromyalgia and step count (mean difference –1.13, 95% CI –1.69 to –0.58, *P<*.001). In the multivariable models adjusting for age, sex, pain level, and the ISS score, the association remained significant (*P=*.01). However, when we added depression symptoms to the model, this association became nonsignificant (*P=*.1), suggesting that depression acts as a major confounder in the association between step count and the presence of fibromyalgia.

## Discussion

In this study, we compared pain level with step count data collected with a smartphone app (Pain-Note). This is the first known study to examine the relationship between step count, measured objectively with a smartphone pedometer, and pain scale in patients with chronic pain. We recruited diverse participants across Japan and obtained real-world data. Using smartphones to obtain step count data enables the data to be easily translated to the general population. Our study shows that a high daily step count is associated with a lower pain scale score except in participants who meet the criteria for fibromyalgia.

One concern of our study is the accuracy of step counts obtained with smartphones. This could be affected both by adherence to use of the smartphone app and by the accuracy of the built-in pedometer itself. A past validation study of adherence to smartphone pedometer use compared step counts derived from a smartphone pedometer and an accelerometer in a free-living test and found that there was an approximately 20% bias between the smartphone and the accelerometer [32]. Therefore, our step count results may be biased by a deviation of, at most, 20% from the true step count. The accuracy of smartphone pedometer step counts has also been investigated, such as in a previous study that showed that a pedometer app was more accurate when data from the built-in pedometer was compared to data from the built-in GPS function [33]. In our study, we used the distance moved (as measured with GPS) in the sensitivity analysis and decided to use only the built-in pedometer in the final analysis. The results obtained with GPS and the pedometer were similar, highlighting the robustness of our procedure. A previous study also reported that adherence to pedometer use was a limitation [15]. Although one-third of our participants disabled the pedometer function during this study, we believe that once they had enabled the pedometer, few of them repeatedly turned the pedometer function on or off during the day. We attribute our relatively low disagreement rate (230 out of 6138) to this. A plausible explanation for why some participants disabled the pedometer function was that they wanted to reduce battery consumption caused by the app working in the background of the smartphone. Therefore, we consider that our study results reflect real-life step counts and that data from the pedometer were complete.

The total daily step count of a typical elderly person in the United States ranges from 2000 to 9000 steps [34]. In a prospective study conducted in Yokohama, Japan, the average daily step counts throughout the year for participants between 40 and 69 years old was 9304 for male participants and 7246 for female participants [35]. In our study, the mean daily step count of the participants was approximately 3000 steps, which is less than half of the previously reported mean daily step count for women in Japan. This discrepancy could be due to the fact that most of the participants in our study had chronic pain; since the Pain-Note app was intended for this population, their step count was lower than that of the general population.

In our study, patients with symptoms of both insomnia and depression had lower walking counts. This result is consistent with previous studies, which have demonstrated that patients with a sleeping disorder or depression participated in fewer daily activities than individuals without these conditions. [36-38] Therefore, a lower daily step count might indicate insomnia or depression; identifying lower step counts might be useful for screening patients with these conditions.

We found, using restricted cubic splines, that there was a noticeable association between higher step count and a lower pain level. Furthermore, the slope of the association was steeper in participants with a smaller step count. Previous studies that have investigated the effect of walking or exercise therapy on pain reduction have assumed a linear association. In fact, the favorable association between increased step count and lower pain level is seen only among patients with chronic pain who do not have fibromyalgia. Participants with fibromyalgia show a different association between step count and pain level. Gracely et al showed that fibromyalgia alters the threshold of pain perception and makes patients more afraid to move [39]. This fear of moving could affect pain level, regardless of step count, but could also help explain the particular increase in pain level we observed in patients with fibromyalgia who had a daily step count below 2000. A meta-analysis of randomized studies with exercise as an intervention found that patients with fibromyalgia reported initial pain when they started to exercise at the beginning of the trials [40]. A previous study also revealed that daily exercise, including walking, reduced pain in fibromyalgia patients [40]. However, there was no association between step count and pain level in patients with fibromyalgia in our study. This may be because the participants who were classified as fulfilling the criteria for fibromyalgia in our study had not been diagnosed and did not have access to proper treatment by health care providers. Furthermore, these patients may not have been informed of the importance of exercise. Previous research suggests that a multidisciplinary approach combining physical exercise and education is important for pain reduction [41-43]. The results from our linear regression analyses, with step count as the outcome and the presence of fibromyalgia as the independent variable, also highlighted why the association between step count and pain level was different in our population. The fact that symptoms of depression are a major confounding variable in the association between fibromyalgia and step count suggests that, as expected, several variables can influence activity level in individuals with this complex syndrome.

This study has several limitations that should be noted. First, our results cannot determine causal associations because the study used a cross-sectional design. Second, there may have been selection biases for age, socioeconomic status, and user characteristics because of the requirement that participant be able to use iOS and the iPhone; in addition, our app is currently available only in the Japanese App Store and in the Japanese language. We also excluded more than 70% of participants who downloaded the app. This large proportion of excluded patients can be explained by the free distribution of our app. We believe that this free distribution means that the accessibility of our app was relatively high, but it also means that a large number of subjects were excluded from the analysis, mainly due to lack of complete data. On the other hand, to improve adherence, we could have distributed the app through a subscription or one-time purchase, but this might have led to additional selection bias, as only participants who could purchase the app would then have been included in the study. Third, our study suffers from self-reporting bias, because the data were collected using self-administered questionnaires. In addition, our analyses related to the presence of fibromyalgia were based on the American College of Rheumatology 2010 criteria, which has been validated in the Japanese language, but has not been validated for use in the form of a smartphone app. Therefore, we must interpret our results as as being obtained from subjects who fulfilled the criteria for fibromyalgia, rather than subjects who had a clinical diagnosis. Finally, step count does not precisely reflect daily physical activity if participants turn off the pedometer function or if they perform activities that do not involve walking, such as swimming or mind-body practices. Considering that previous research has found a 20% bias in smartphone-based step trackers, the step count recorded by our app might have been an underestimate by as much as 20%.

Future research might include a longitudinal study to further explore the causal relationship between step count and pain. Analyzing predictors of the development or improvement of chronic pain would allow the planning of notification strategies or interventions in high-risk populations to reduce the burden of chronic pain.

In conclusion, our study revealed a significant association between high step count and low pain level among participants who did not meet criteria for fibromyalgia. In data that were stratified for patients with and without fibromyalgia, the likelihood test of a spline model did not show statistical significance; this could be the result of a lack of power, since the association was nonlinear in the overall sample. The characteristic shape of the association between patients with fibromyalgia and those without fibromyalgia might reflect different mechanisms of physical movement and pain perception. To find the causal association between step count and pain level, future studies should be designed to obtain longitudinal data and include participants from other countries who speak different languages; this would broaden the generalizability of our findings to a more diverse population. The extensive health care data that can be obtained with our Pain-Note app may ultimately help raise awareness regarding the importance of daily activity for preventing chronic pain.

## Appendix

Multimedia Appendix 1 Flow chart of data collection in Pain-Note app and results of sensitivity analysis.

## Abbreviations

ISS: insomnia severity scale
SS: symptom severity
WPI: widespread pain index
