# Supplementary material for: Association Between Step Count Measured With a Smartphone App (Pain-Note) and Pain Level in Patients With Chronic Pain: Observational Study
Source: JMIR Form Res. 2022 Apr 6;6(4):e23657. doi: 10.2196/23657 (PMC9021942; doi:10.2196/23657)

APPENDIX

Appendix1 Questionnaire for depressive symptoms

1. Do you enjoy your life?
2. Do you enjoy what have you done before as same as before?
3. Do you feel tired when you do something you could easily before?
4. Do you feel as good as other people?
5. Do you think about death?
6. Do you feel so depressed that you think about suicide?
7. Do you have something very difficult or painful especially these days?
8. Do you feel like eating?
9. Do you feel depressed?

Appendix flow chart of the study questionnaire on the Pain-note app.

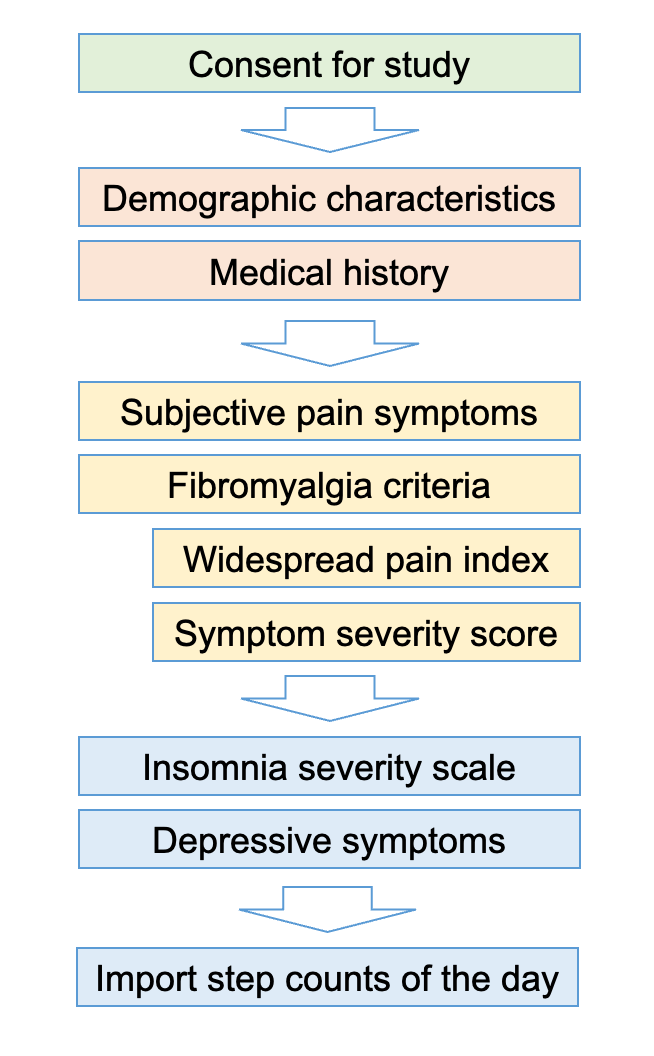


Association between moving distance and pain scale using restricted cubic spline in all participants.


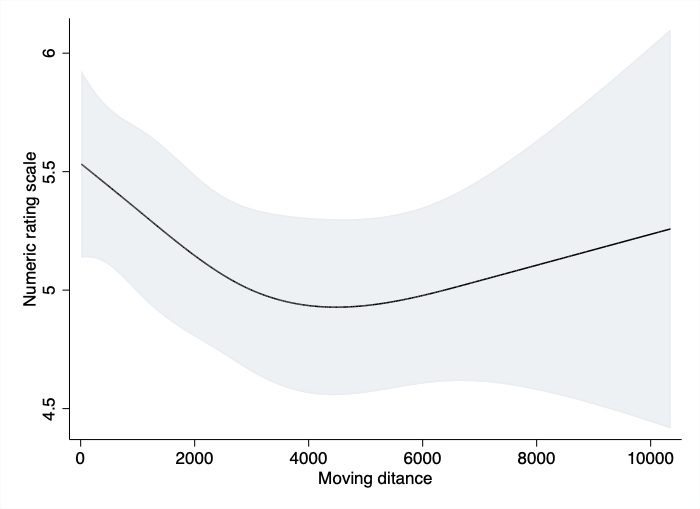


Association between moving distance and pain scale using restricted cubic spline in fibromyalgia population.


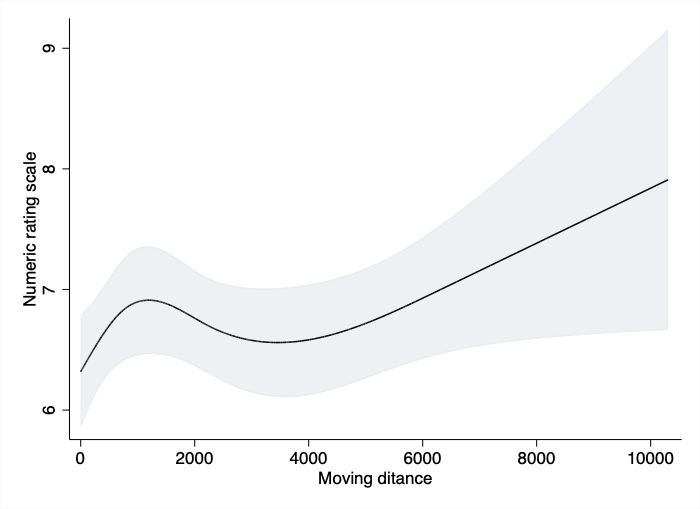


Association between moving distance and pain scale using restricted cubic spline in non-fibromyalgia population.


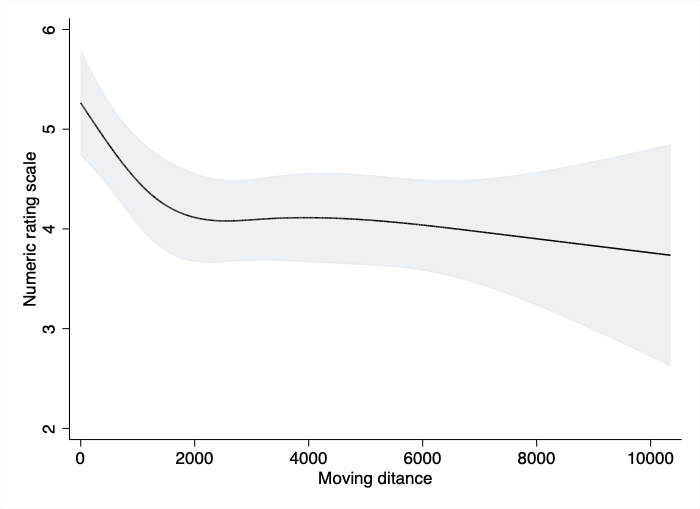

Supplement: Multimedia Appendix 1 [file formative_v6i4e23657_app1.docx]
